# Supplementary material for: High-intensity training induces non-stoichiometric changes in the mitochondrial proteome of human skeletal muscle without reorganisation of respiratory chain content
Source: Nat Commun. 2021 Dec 3;12:7056. doi: 10.1038/s41467-021-27153-3 (PMC8642543; doi:10.1038/s41467-021-27153-3)
Supplement: Supplementary file 1 — Supplementary Information [file 41467_2021_27153_MOESM1_ESM.pdf]

Supplementary information for:

High-intensity training causes non-stoichiometric changes in the mitochondrial proteome of human skeletal muscle without reorganisation of respiratory chain content

Cesare Granata[1,2,9,†,*], Nikeisha J. Caruana[1,3,†], Javier Botella[1], Nicholas A. Jamnick[1,4], Kevin Huynh[5], Jujiao Kuang[1], Hans A. Janssen[1], Boris Reljic[3,10], Natalie A. Mellett[5], Adrienne Laskowski[2], Tegan L. Stait[6], Ann E. Frazier[6,7], Melinda T. Coughlan[2,5], Peter J. Meikle[5], David R. Thorburn[6,7,8], David A. Stroud[3,6,‡,*] and David J. Bishop[1,‡,*]

[1]Institute for Health and Sport (iHeS), Victoria University, Melbourne, VIC, 3011, Australia

[2]Department of Diabetes, Central Clinical School, Monash University, Melbourne, VIC, 3004, Australia

[3]Department of Biochemistry and Pharmacology and Bio21 Molecular Science and Biotechnology Institute, The University of Melbourne, Parkville, VIC, 3010, Australia

[4]Metabolic Research Unit, School of Medicine and Institute for Mental and Physical Health and Clinical Translation (iMPACT), Deakin University, Geelong, VIC, Australia

[5]Baker Heart & Diabetes Institute, Melbourne, VIC, 3004, Australia

[6]Murdoch Children's Research Institute, Royal Children's Hospital, Melbourne, VIC, 3052, Australia

[7]Department of Paediatrics, The University of Melbourne, Melbourne, VIC, 3052, Australia

[8]Victorian Clinical Genetics Services, Royal Children's Hospital, Melbourne, VIC, 3052, Australia

[9]Current address: Institute for Clinical Diabetology, German Diabetes Center, Leibniz Center for Diabetes Research, Heinrich Heine University, 40225, Düsseldorf, Germany

[10]Current address: Department of Biochemistry and Molecular Biology, Monash Biomedicine Discovery Institute, Monash University, 3800, Melbourne, Australia.

[†]These authors contributed equally

[‡]These authors jointly supervised this work

******Correspondence***:** cesare.granata@monash.edu (C.G.), david.stroud@unimelb.edu.au (D.A.S), david.bishop@vu.edu.au (D.J.B)

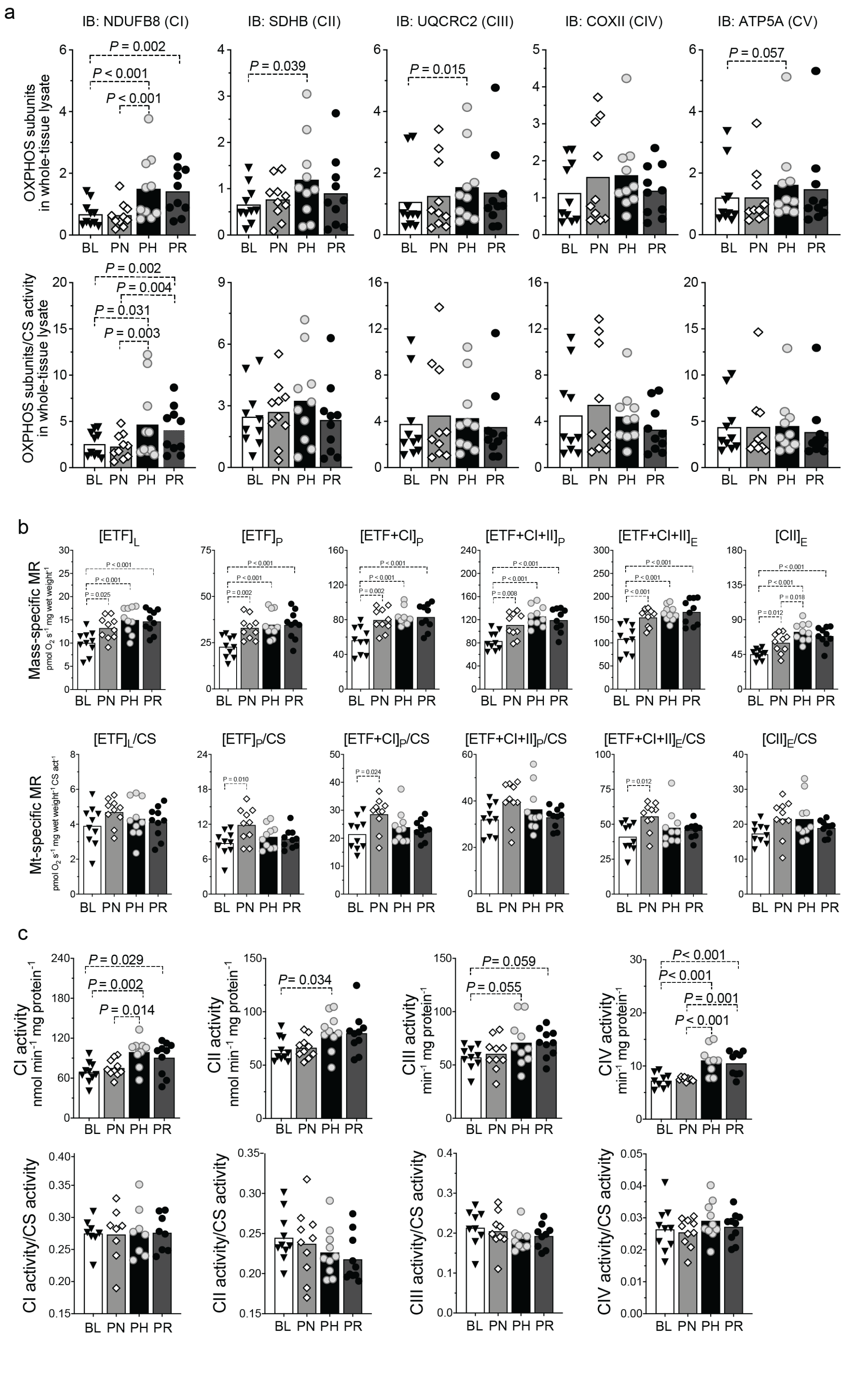

Supplementary Fig. 1. a, Top panels: protein content of selected subunits of oxidative phosphorylation (OXPHOS) complexes by immunoblotting in whole-tissue (vastus lateralis) homogenates; lower panels: values from top panels normalised by citrate synthase (CS)

activity (obtained in Fig. 1d). All samples analysed were derived from the same experiment and blots were processed in parallel. **b**, Top panels: mass-specific mitochondrial respiration (MR) in permeabilised human vastus lateralis muscle fibres measured with the following substrate-uncoupler-inhibitor titration (SUIT) protocol: $[ETF]_L$, leak respiration state (L) in absence of adenylates and electron input through ETF; $[ETF]_P$, maximal OXPHOS state (P) with electron input through ETF; $[ETF+CI]_P$, P with convergent electron input through ETF + CI; $[ETF+CI+II]_P$, P with convergent electron input through ETF + CI + CII; $[ETF+CI+II]_E$, maximal electron transport chain capacity (E) with convergent electron input through ETF + CI + CII; $[CII]_E$, E with electron input through CII. Lower panels: mitochondrial (mt)-specific MR obtained by normalising values of mass-specific MR by CS activity (obtained in Fig. 1d). **c**, Top panels: enzymatic activity of electron transport chain (ETC) complexes in whole-tissue (vastus lateralis) homogenates; lower panels: values from top panels normalised by CS activity (obtained in Fig. 1d). Source data for all panels are provided as a Source Data file. BL: baseline; PN: post-NVT; PH: post-HVT; PR: post-RVT; CI-V: complex I to V; IB: immunoblotting; filled triangles, empty diamonds, as well as empty and filled circles represent individual values; bars represent mean values; n = 10 for all analyses. All datasets analysed by repeated measures one-way ANOVA followed by Tukey's post hoc testing, except for $[ETF+CI+II]_P/CS$, which was analysed by Friedman test followed by Dunn's post hoc testing, as not normally distributed; $P < 0.05$.

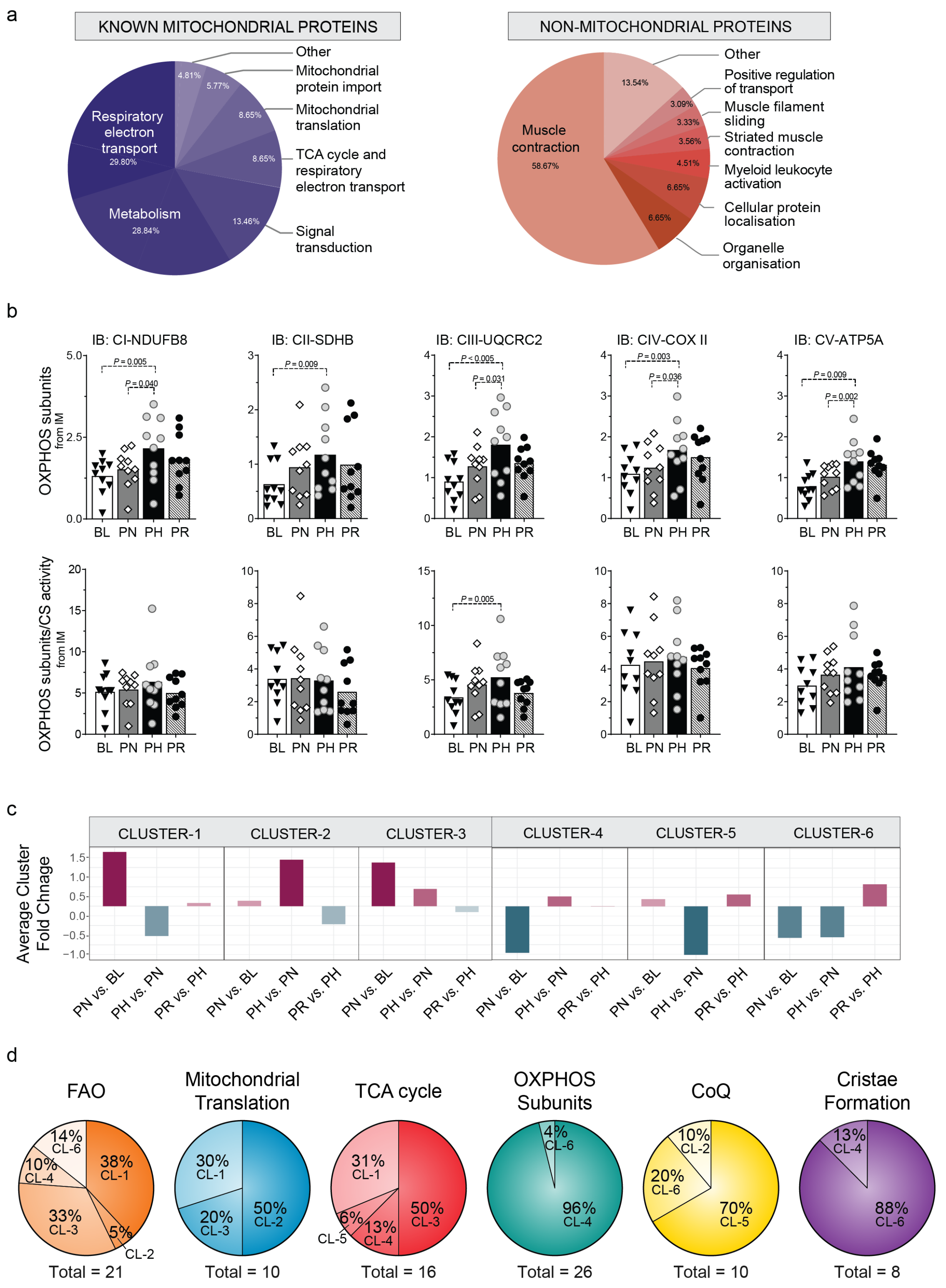

Supplementary Fig. 2. a, Pie charts showing the relative enrichment terms, as determined by *Reactome*, of "Known Mitochondrial" and "non-mitochondrial" proteins, identified by the Integrated Mitochondrial Protein Index (IMPI) database[28] from isolated mitochondria (IM) fractions. **b**, Top panels: training-induced changes in protein content of selected subunits of

oxidative phosphorylation (OXPHOS) complexes by SDS-PAGE in IM fractions from vastus lateralis muscle biopsies; lower panels: values from top panels normalised by CS activity (obtained in Fig. 1d). Source data are provided as a Source Data file; filled triangles, empty diamonds, and empty and filled circles represent individual values; bars represent mean values. All samples analysed were derived from the same experiment and blots were processed in parallel. **c**, Profile plots of the scaled expression mean of proteins within the six clusters as determined in Fig. 2e. **d**, Venn diagram representation of the protein distribution from each of the six main protein functional classes (as determined in Supplementary Data 6) within the six clusters as determined in Fig. 2e. BL: baseline; PN: post-NVT; PH: post-HVT; PR: post-RVT; IB: immunoblotting; n = 10 for all analyses; all datasets analysed by repeated measures one-way ANOVA followed by Tukey's post hoc testing; $P < 0.05$.

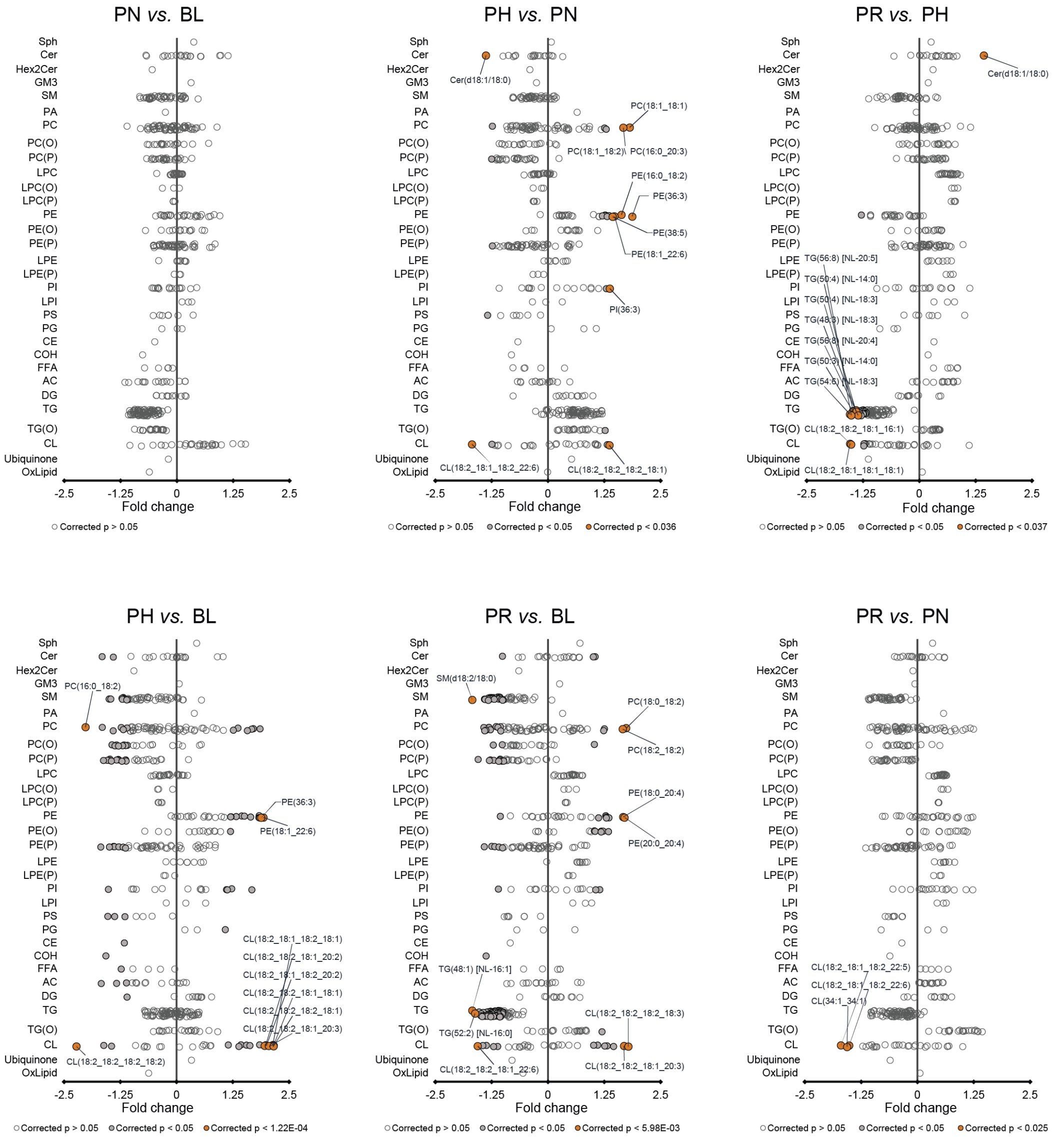

Supplementary Fig. 3. Forest plots displaying training-induced changes in individual lipid species between pairs of time points obtained from lipidomics analysis of isolated mitochondria (IM) fractions from human vastus lateralis muscle biopsies; n = 10 for all analyses; all data from differential expression tests taken from *Limma* as determined by linear modelling using empirical Bayes moderation as per the methods and displayed as fold change; open circles show non-significant species, grey circles show species with $P < 0.05$ after correction for multiple comparisons (Benjamini-Hochberg). The top 10 species after correction for multiple comparisons are highlighted with orange circles. BL: baseline; PN: post-NVT; PH: post-HVT; PR: post-RVT.
