# Supplementary material for: High-intensity training induces non-stoichiometric changes in the mitochondrial proteome of human skeletal muscle without reorganisation of respiratory chain content
Source: Nat Commun. 2021 Dec 3;12:7056. doi: 10.1038/s41467-021-27153-3 (PMC8642543; doi:10.1038/s41467-021-27153-3)
Supplement: Supplementary file 3 — Description of Additional Supplementary Files [file 41467_2021_27153_MOESM3_ESM.pdf]

Description of Additional Supplementary Files

File name: Supplementary Data 1

Description: Raw and processed transcriptomic data. Raw RNA-seq counts were z-scored and transcripts annotated according to mitochondrial pathway. Further detail provided in the legend tab of the linked Excel file.

File name: Supplementary Data 2

Description: Differentially expressed transcripts and pathway enrichment. Differentially expressed transcripts were determined for each sequential training step as described in the methods. Gene ontology biological process pathway enrichment was performed on differentially expressed transcripts as described in the methods. Further detail provided in the legend tab of the linked Excel file.

File name: Supplementary Data 3

Description: Raw mitochondrial proteomics data. Raw label free quantification (LFQ) proteomics data provided in linear (non log) space. LFQ intensities were used, along with mitochondrial annotation for each protein, to calculate mitochondrial protein enrichment (MPE) as described in the methods. Further detail provided in the legend tab of the linked Excel file.

File name: Supplementary Data 4

Description: Normalised mitochondrial proteomics data. BESt normalisation was performed on raw proteomics data as described in the methods. Data is presented in linear and scaled formats. Further detail provided in the legend tab of the linked Excel file.

File name: Supplementary Data 5

Description: Content-normalised changes to the mitochondrial proteome can be attributed to six defined clusters. Differentially expressed proteins across each training phase were identified from normalised proteomics data and relative changes clustered using hierarchical clustering. Pathways underpinning each cluster were identified using gene ontology biological process enrichment. Further detail provided in the legend tab of the linked Excel file.

File name: Supplementary Data 6

Description: Protein functional classes and metabolic pathways. Description of enriched protein functional classes and metabolic pathways, which were generated according to Reactome pathways and/or from literature searches (complete details in the Methods section). Further detail provided in the legend tab of the linked Excel file.

File name: Supplementary Data 7

Description: Differentially expressed proteins between time points and grouped on a per “protein functional classes and metabolic pathways” basis. Differentially expressed proteins were determined by matching the differentially expressed proteins identified by direct comparison between each pair of time points (by two-sided t-test) and the 185 differentially expressed proteins identified across the three

training phases determined by linear model fit using limma with empirical Bayes method. An overview of the differentially expressed proteins obtained by the above matching is given for each protein functional class and metabolic pathway. Further detail provided in the legend tab of the linked Excel file.

File name: Supplementary Data 8

Description: Raw and processed lipidomics data. Raw lipid concentrations for both species and totals are provided. Lipid concentrations for both species and overall total were normalised by total cardiolipin content. Scaled averages of each species is also given. Further detail provided in the legend tab of the linked Excel file.

File name: Supplementary Data 9

Description: Differentially expressed lipid species. Differentially expressed lipid species were determined for each sequential training step as described in the methods. Further detail provided in the legend tab of the linked Excel file.
