# Supplementary material for: High-intensity training induces non-stoichiometric changes in the mitochondrial proteome of human skeletal muscle without reorganisation of respiratory chain content
Source: Nat Commun. 2021 Dec 3;12:7056. doi: 10.1038/s41467-021-27153-3 (PMC8642543; doi:10.1038/s41467-021-27153-3)
Supplement: Supplementary file 13 — Reporting Summary [file 41467_2021_27153_MOESM13_ESM.pdf]

nature research

Corresponding author(s): Prof. David Bishop, Dr. David Stroud, Dr. Cesare Granata

Last updated by author(s): Oct 21, 2021

Reporting Summary

Nature Research wishes to improve the reproducibility of the work that we publish. This form provides structure for consistency and transparency in reporting. For further information on Nature Research policies, see our Editorial Policies and the Editorial Policy Checklist.

Statistics

For all statistical analyses, confirm that the following items are present in the figure legend, table legend, main text, or Methods section.

n/a	Confirmed	
☐	☒	The exact sample size (*n*) for each experimental group/condition, given as a discrete number and unit of measurement
☐	☒	A statement on whether measurements were taken from distinct samples or whether the same sample was measured repeatedly
☐	☒	The statistical test(s) used AND whether they are one- or two-sided<br>*Only common tests should be described solely by name; describe more complex techniques in the Methods section.*
☒	☐	A description of all covariates tested
☐	☒	A description of any assumptions or corrections, such as tests of normality and adjustment for multiple comparisons
☐	☒	A full description of the statistical parameters including central tendency (e.g. means) or other basic estimates (e.g. regression coefficient) AND variation (e.g. standard deviation) or associated estimates of uncertainty (e.g. confidence intervals)
☐	☒	For null hypothesis testing, the test statistic (e.g. *F*, *t*, *r*) with confidence intervals, effect sizes, degrees of freedom and *P* value noted<br>*Give P values as exact values whenever suitable.*
☒	☐	For Bayesian analysis, information on the choice of priors and Markov chain Monte Carlo settings
☒	☐	For hierarchical and complex designs, identification of the appropriate level for tests and full reporting of outcomes
☒	☐	Estimates of effect sizes (e.g. Cohen's *d*, Pearson's *r*), indicating how they were calculated

Our web collection on statistics for biologists contains articles on many of the points above.

Software and code

Policy information about availability of computer code

Data collection	STAR aligner (v2.5.3a), MaxQuant (v1.6.1.0), Agilent Mass Hunter (vB.09.00)
Data analysis	Bio-Rad Image Lab 5.0, Cytoscape v3.7.1, Prism v8.4.2, R v3.6.3; packages: Limma,imputeLCMD, Enrichr, edgeR, Adobe Illustrator (CC2018.22.1), R scripts detailed in the code availability section available at https://doi.org/10.5281/zenodo.5576974.

For manuscripts utilizing custom algorithms or software that are central to the research but not yet described in published literature, software must be made available to editors and reviewers. We strongly encourage code deposition in a community repository (e.g. GitHub). See the Nature Research guidelines for submitting code & software for further information.

Data

Policy information about availability of data

All manuscripts must include a data availability statement. This statement should provide the following information, where applicable:

- Accession codes, unique identifiers, or web links for publicly available datasets
- A list of figures that have associated raw data
- A description of any restrictions on data availability

The mass spectrometry proteomics data has been deposited in the ProteomeXchange Consortium via the PRIDE partner repository under accession code PXD026219 (http://proteomecentral.proteomexchange.org/cgi/GetDataset?ID=PXD026219). The transcriptomic data has been deposited in the NCBI and can be found under the BioProject PRJNA732106 (https://www.ncbi.nlm.nih.gov/bioproject/?term=PRJNA732106). Lipidomics data has been deposited in the NIH Common Fund's National Metabolomics Data Repository (NMDR) website, the Metabolomics Workbench, under Project ID ST001907 (http://dx.doi.org/10.21228/M8G69Q). Figures 1, 2, 3, 4, 5, S2 and S3, make use of the associated raw data after data analysis was performed.

Field-specific reporting

Please select the one below that is the best fit for your research. If you are not sure, read the appropriate sections before making your selection.

☒ Life sciences ☐ Behavioural & social sciences ☐ Ecological, evolutionary & environmental sciences

For a reference copy of the document with all sections, see nature.com/documents/nr-reporting-summary-flat.pdf

Life sciences study design

All studies must disclose on these points even when the disclosure is negative.

Sample size	We analyzed muscle samples from ten healthy male participants. Muscle samples were analysed before and after each of the three training phases. Our study included n=10 for all biological analyses, as well as for the proteomics and lipidomics; however, due to limitations with the amounts of skeletal muscle biopsy material obtained from the participants, n=5 was used for the transcriptome analysis.<br><br>The power calculation was based on the ability to detect changes in mitochondrial respiration and for the omics measurements, which were primary endpoints for our research.<br>The initial power calculation was based on previously published values of changes in mitochondrial respiration obtained from a study with a similar design conducted in our laboratory (Granata et al. 2016 - PMID: 27402675). In this study, mitochondrial respiration increased from 65.3 ± 8.0 to 96.3 ± 13.3 (ES = 2.9), following 3 weeks of high-volume training (HVT), and decreased from 96.3 ± 13.3 to 79.3 ± 12.8 (ES = 1.3) following 2 weeks of reduced training (RVT). Using an alpha of 0.05, and a desired power of at least 0.80, the total effective sample size necessary to achieve statistical significance would be 4 and 7 participants for the HVT and RVT phase, respectively. To cover for potential drop outs, the final number of participants was increased by 30% to n = 10. This is in line with published research in the field using sample sizes between 7 and 11 participants (Tonkonogi et al., 2000 - PMID: 11034627; Walsh et al., 2001 - PMID: 11484774; Daussin et al., 2008 - PMID: 18417645; Granata et al., 2016 - PMID: 27402675; Vincent et al., 2015 - PMID: 25759671; Zisko et al., 2015 - PMID: 25969700). With respect to omics measurements, it has previously been demonstrated that n = 6 is a sufficient sample size for omics experiments (Hogrebe et al., 2018 - PMID: 29535314). While the transcriptomics portion of our study was performed on n = 5, due to limitations in tissue availability, the results were largely negative as described in the manuscript and were not used to inform the major conclusions.
Data exclusions	No data was excluded
Replication	Due to the limited availability of biopsy material and the use of this material to conduct multiple LCMS experiments, as well as the limitations on the LCMS instrumentation time, all experiments were performed only once for the 10 subjects for PRE and POST conditions (including each of the normal, high and reduced training phases) . We verified the reproducibility of all analyses using a multidimensional scaling analysis (MDS) as well the number of peptide and protein identifications for each replicate. The attempts that were used for checking the reproducibility this way were successful.
Randomization	Randomization was applied for the study.
Blinding	Yes, the investigators were blinded to the group.

Reporting for specific materials, systems and methods

We require information from authors about some types of materials, experimental systems and methods used in many studies. Here, indicate whether each material, system or method listed is relevant to your study. If you are not sure if a list item applies to your research, read the appropriate section before selecting a response.

Materials & experimental systems

n/a	Involved in the study	
☐	☒	Antibodies
☒	☐	Eukaryotic cell lines
☒	☐	Palaeontology and archaeology
☒	☐	Animals and other organisms
☐	☒	Human research participants
☒	☐	Clinical data
☒	☐	Dual use research of concern

Methods

n/a	Involved in the study	
☒	☐	ChIP-seq
☒	☐	Flow cytometry
☒	☐	MRI-based neuroimaging

Antibodies

Antibodies used	OXPHOS cocktail, Abcam, Cat#ab110411; RRID:AB_2756818<br>Anti-NDUFB8, Abcam, Cat.#ab110242; RRID:AB_10859122<br>Anti-NDUFA9, Abcam, Cat.#ab14713; RRID:AB_301431

Anti-UQCRC2, Abcam, Cat.#ab14745; RRID:AB_2213640
Anti-COXIV, Abcam, Cat. #ab14744; RRID:AB_301443

Validation

Total OXPHOS Human WB Antibody Cocktail, Abcam, Cat#ab110411; RRID:AB_2756818
This ab cocktail consists of five very specific monoclonal antibodies directed against different subunits of the five OXPHOS complexes in human mitochondria. We have used this antibody in the following publication with expected results: Stroud et al. 2016 Nature; https://doi.org/10.1038/nature19754. In addition, the manufacturer has 290 citations for this antibody on their website: https://www.abcam.com/total-oxphos-human-wb-antibody-cocktail-ab110411.html

Anti-NDUFB8, Abcam, Cat.#ab110242; RRID:AB_10859122
We have previously verified this antibody using a human knockout cell line for NDUFB8 (Stroud et al. 2016 Nature; https://doi.org/10.1038/nature19754). In addition, the manufacturer has 158 citations for this antibody on their website: https://www.abcam.com/ndufb8-antibody-20e9dh10c12-ab110242.html

Anti-NDUFA9, Abcam, Cat.#ab14713; RRID:AB_301431
The manufacturer has validated this using a human knockout cell line for NDUFA9 and has 173 citations for this antibody on their website:
https://www.abcam.com/ndufa9-antibody-20c11b11b11-ab14713.html

Anti-UQCRC2, Abcam, Cat.#ab14745; RRID:AB_2213640
The manufacturer has validated this antibody using imaging (showing mitochondrial localisation in human cells) and through SDS-PAGE by molecular weight. The antibody has 195 citations listed on their website: https://www.abcam.com/uqcrc2-antibody-13g12af12bb11-ab14745.html

Anti-COXIV, Abcam, Cat. #ab14744; RRID:AB_301443
We have used this antibody in the following publication with expected results: Stroud et al. 2016 Nature; https://doi.org/10.1038/nature19754. The manufacturer has 246 citations for this antibody on their website: https://www.abcam.com/cox-iv-antibody-20e8c12-ab14744.html

Human research participants

Policy information about studies involving human research participants

Population characteristics

Young (22.3 ± 5.2 years), lean (BMI: 24.8 ± 2.9 [kg/m2]), untrained (VO2peak: 46.7 ± 8.2 [mL min-1 kg-1] and healthy male subjects (n = 10) were studied.

Recruitment

Participants were recruited from the Melbourne area in Victoria, Australia, by advertising on the University campus noticeboards and the surrounding areas (Footscray), and by presenting the study before University classes without bias toward a particular department or faculty (Footscray Park Campus). Other than the above, self selection bias was not controlled; however, we believe the impact to be minimal as the above population characteristics for untrained individuals were applied. The participants gave their written, informed consent to participate in the study. Approval for the study procedures, which confirmed to the standards set by the latest revision of the Declaration of Helsinki, was granted by the Victoria University Research Ethics Committee (HRE15-126). Recruitment was based on the following inclusion and exclusion criteria: Inclusion criteria were:
Ages 18-35 years.
Moderately-trained (less than 4 h per week of unstructured aerobic activity for half a year prior to the study).
Not regularly engaged in cycling-based sports.
Healthy males, non-smokers, not taking medication.
BMI between 21 and 27 kg/m2
Exclusion criteria were:
Females
Smokers
Males younger than 18 or older than 35
BMI less than 22 or more than 27
Conditions that may have precluded their participation: eg. cardiovascular, musculoskeletal and/or metabolic problems.

Ethics oversight

Victoria University Human Research Ethics Committee (HRE15-126)

Note that full information on the approval of the study protocol must also be provided in the manuscript.
