# Supplementary material for: High-intensity training induces non-stoichiometric changes in the mitochondrial proteome of human skeletal muscle without reorganisation of respiratory chain content
Source: Nat Commun. 2021 Dec 3;12:7056. doi: 10.1038/s41467-021-27153-3 (PMC8642543; doi:10.1038/s41467-021-27153-3)

Fig. 5a and b - uncropped images w/ size markers in blue

Membranes were imaged in their entirety, except for membrane 4 of CI and CII, which were cut vertically along the 11th well; red boxes indicate the lane used for quantitation for each specific antibody; black vertical boxes represent the cropped lanes used to generate Fig. 5a

IB: NDUFA9 (CI) from antibody ab14713

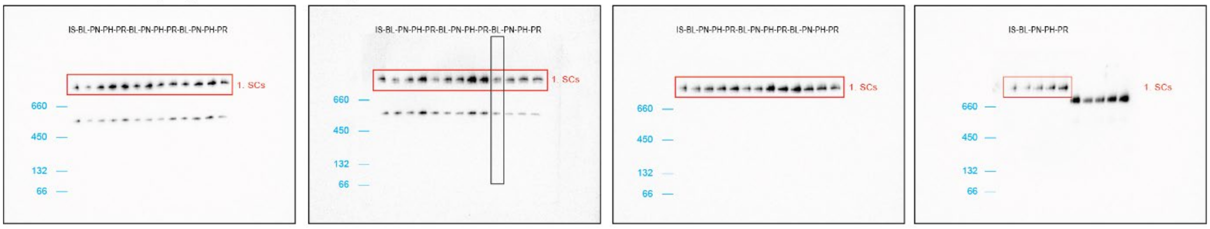

IB: UQCRC2 (CIII) from antibody ab14745

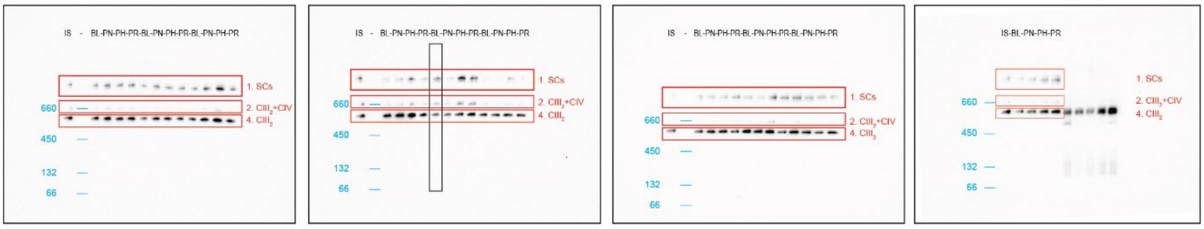

IB: COXIV (CIV) from antibody ab14744

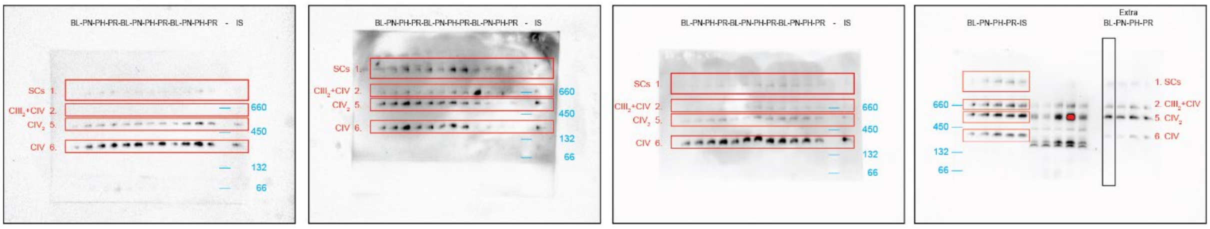

Supp. Fig. 1a - uncropped images w/ size markers in blue
All membranes blotted with ab110411 were cut at ~75 kDa prior to imaging; those blotted with ab110242 were imaged intact; red boxes indicate the lane used for quantitation for each specific antibody.

IB: NDUFB8 (CI) from NDFUB8 antibody (ab110242) first 3 images, & total OXPHOS antibody (ab110411) last image

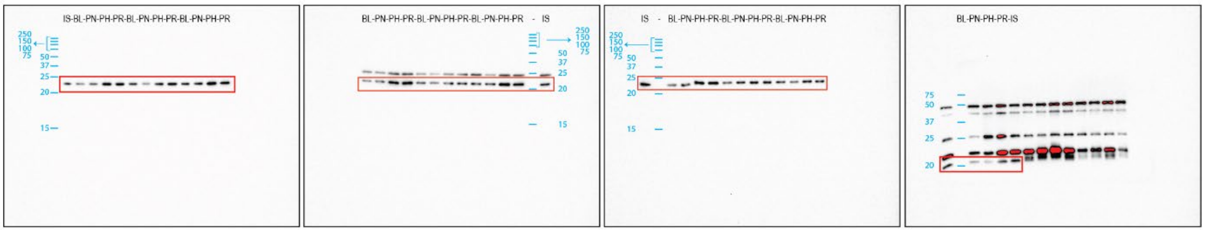

IB: SDHB (CII) from total OXPHOS antibody (ab110411)

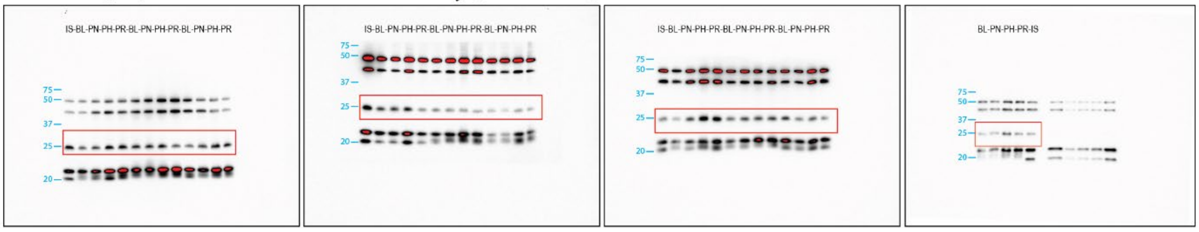

IB: UQCRC2 (CIII) from total OXPHOS antibody (ab110411)

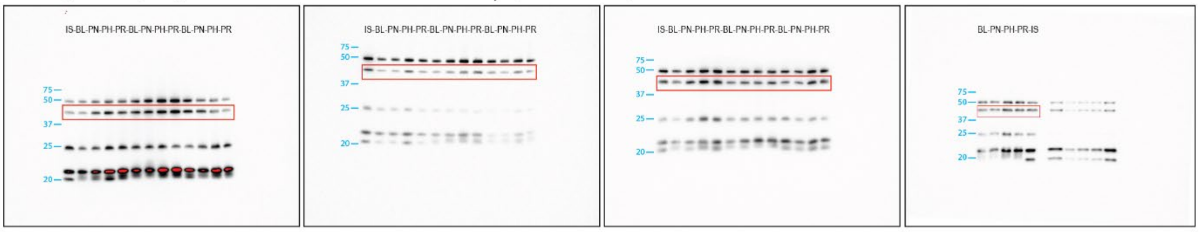

IB: COXII (CIV) from total OXPHOS antibody (ab110411)

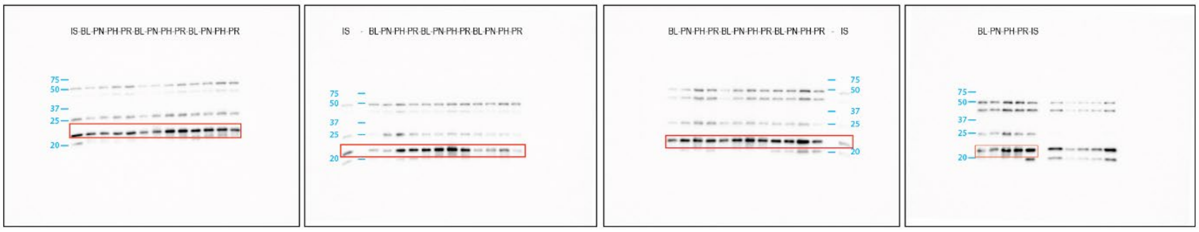

IB: ATP5A (CV) from total OXPHOS antibody (ab110411)

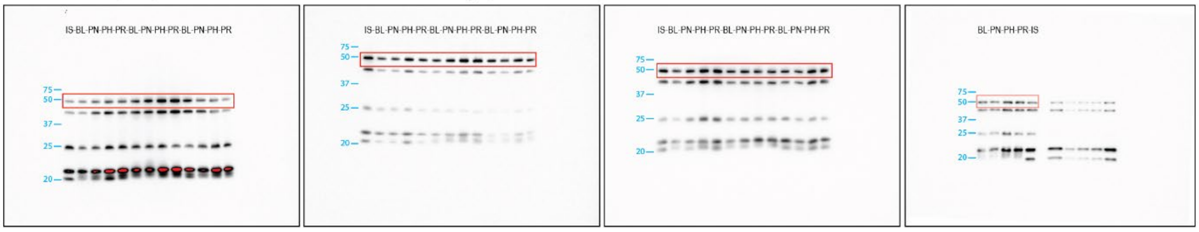

Supp. Fig. 2b - uncropped images w/ size markers in blue
Membranes were cut at ~75 kDa prior to imaging; ; red boxes indicate the lane used for quantitation for each specific antibody.

IB: NDUFB8 (CI) from total OXPHOS antibody (ab110411)

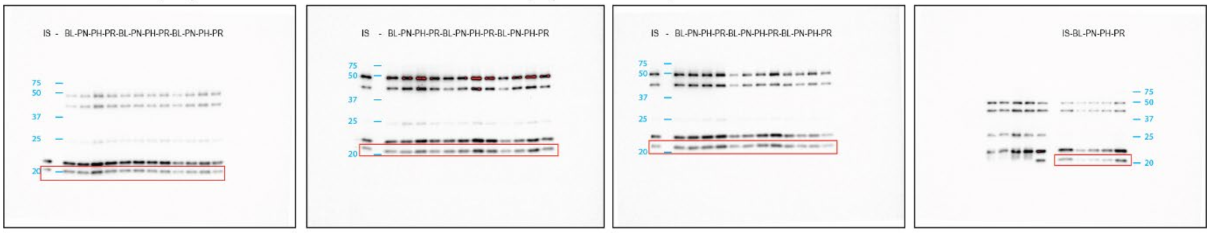

IB: SDHB (CII) from total OXPHOS antibody (ab110411)

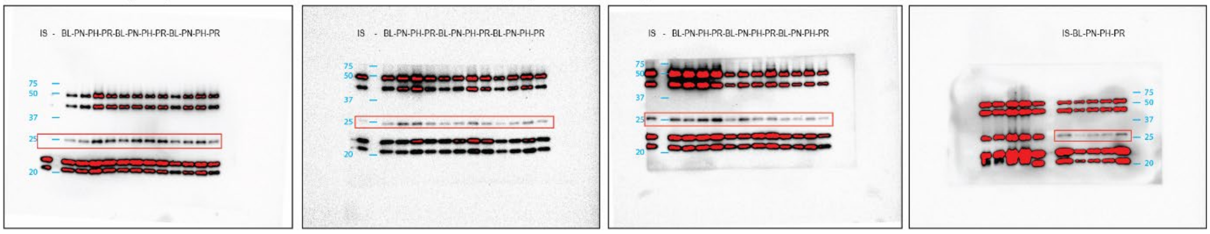

IB: UQCRC2 (CIII) from total OXPHOS antibody (ab110411)

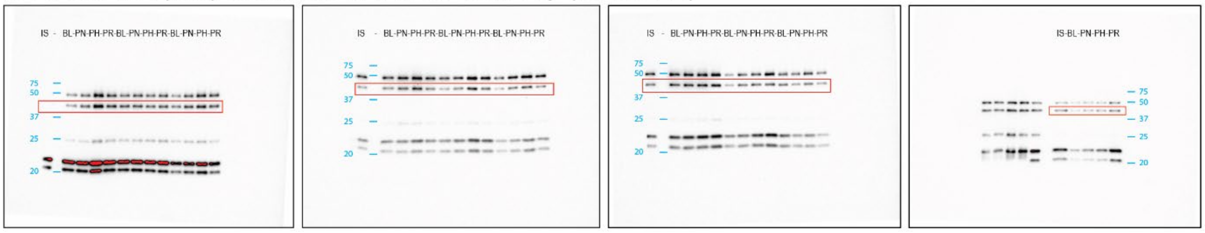

IB: COXII (CIV) from total OXPHOS antibody (ab110411)

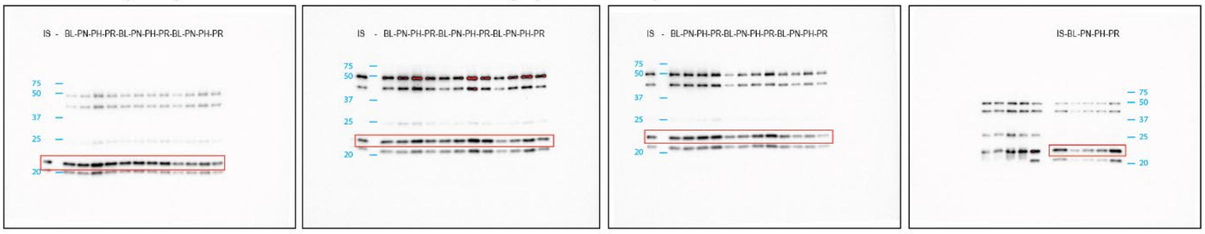

IB: ATP5A (CV) from total OXPHOS antibody (ab110411)

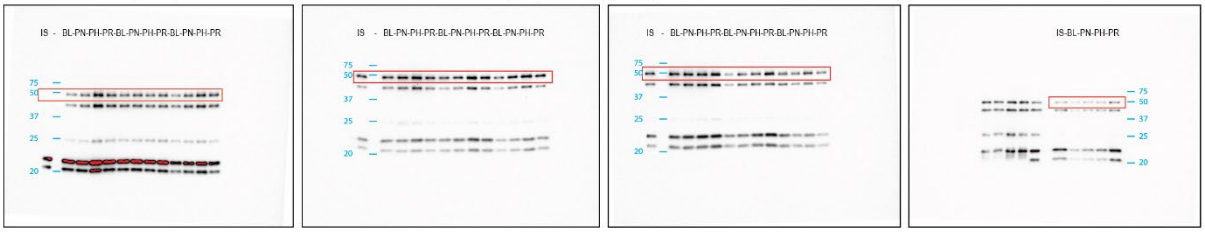

Supplement: Supplementary file 14 — Source Data [file 41467_2021_27153_MOESM14_ESM.zip › Source Data uncropped blots.pdf]
